# Functional Rabbit Syndrome: A Case Report

**DOI:** 10.5334/tohm.674

**Published:** 2021-12-30

**Authors:** Maria Stella Aniello, Sergio Altomare, Pasquale Difazio, Maurizio Giorelli

**Affiliations:** 1 Operative Unit of Neurology, “Dimiccoli” General Hospital, Barletta, ASL BT, Italy; 2 Operative Unit of Nuclear Medicine, “Dimiccoli” General Hospital, Barletta, ASL BT, Italy

**Keywords:** tremor, functional, hyperkinetic movement disorder, rabbit syndrome

## Abstract

Rabbit Syndrome is a rare involuntary movement occurring in 1.5–4.4% of patients receiving antipsychotics and characterized by rapid, regular movements (4–6 Hz) of the oral and masticatory musculature resembling the chewing motions of a rabbit. Herein we describe a middle-aged woman who presented with a rabbit syndrome characterized by several clues of psychogenicity such as sudden onset, distractibility, variability and complete “miracolous” remission.

## Background

Rabbit Syndrome is a rare involuntary movement occurring in 1.5–4.4% of patients receiving antipsychotics and characterized by rapid, regular movements (4–6 Hz) of the oral and masticatory musculature resembling the chewing motions of a rabbit [1,2]. A 65 year old woman presented to the ED complaining of sudden onset of a chin tremor in the few days prior to presentation. She complained that the tremor disappeared during sleep. Her family history was remarkable for juvenile onset of hand tremor in paternal aunts and the patient herself complained of an intermittent increase in the physiological tremor of her hands. Her personal medical history is characterized by partial removal of meningioma from the left temporal hollow more than 20 years ago. She has never taken neuroleptics but she is taking Pregabalin 300 mg b.i.d. from several years to treat an atypical, post-surgical facial pain lasting from resection of meningioma. Her personal medical history ruled out previous psychiatric disturb but a history of juvenile familial isolation and suffering for death of her parents in the early years of her life.

## Phenomenology shown

At admission in the Neurological Unit, the patient presented with continuous, rhythmic, vertical movements of her lips and masticatory muscles resembling those of a rabbit chewing characterized by high frequency per second and amplitude. Movements were severely challenging and disabling for the patient herself. Nevertheless, when invited to perform simultaneous hand tasks at variable frequency, the movements of the mouth presented with the same variability of the voluntary exercises performed by the hands (entrainment phenomenon, ***segment 1***). Movements decreased when performing complex patterns of finger opposition (distractibility, ***segment 2***) and completely disappeared while talking (variability and incosistency, ***segment 3***). Being aware that a rabbit syndrome can be the unusual presentation of organic diseases [4], a [(123)I]β-CIT SPECT was performed and reported as negative for dopaminergic denervation of basal ganglia and a brain MRI showed persistence of the known residual meningioma in the left temporal fossa but without any other pathological sign. Finally, the movements completely disappeared (***segment 4***) even at rest after having a brief conversation with the patient herself underlying the relevance of negativity of the work up performed and critically discussing about inconsistency of the symptoms she manifested. A diagnosis of functional movement disorder presenting as rabbit syndrome was made on the basis of history and positive exam findings as noted above.

**Video:** **Segment 1** entrainment phenomenon. The movements of the mouth followed the same variability of the voluntary exercises performed by the hands when the patient was invited to perform hand tasks at variable frequency. **Segment 2** distractibility and variability. Rabbit movements of the mouth decreased when performing complex patterns of finger opposition. **Segment 3** variability and inconsistency. Rabbit syndrome completely disappeared while talking. **Segment 4** sudden and unjustified disappearance of rabbit syndrome after knowing results from work up and ensuring on health condition.

## Educational value

Diagnosis of functional movement disorders should not be derived by exclusion of organic disease but rather, by demonstration of “red flags” (positive exam findings) suggestive of a functional disorder. Sudden onset of symptoms, inconsistency, incongruence, entrainment of frequency, variability of speed, amplitude, and frequency, and distractibility, are all signs which should be carefully investigated while dealing with a functional movement disorder [3]. Attention to anamnestic clues such as models of disease in relatives and tendency to develop somatoform symptoms can also be helpful as associative contributors.
